# Association Between Trajectory of Severe Hypoglycemia and Dementia in Patients With Type 2 Diabetes: A Population-based Study

**DOI:** 10.2188/jea.JE20200518

**Published:** 2022-09-05

**Authors:** Chung-Yi Li, Chia-Lun Kuo, Ya-Hui Chang, Chin-Li Lu, Santi Martini, Wen-Hsuan Hou

**Affiliations:** 1 Department of Public Health, College of Medicine, National Cheng Kung University, Tainan, Taiwan; 2 Department of Epidemiology, Faculty of Public Health, Universitas Airlangga, Surabaya, Indonesia; 3 Department of Public Health, College of Public Health, China Medical University, Taichung, Taiwan; 4 Department of Healthcare Administration, College of Medical and Health Science, Asia University, Taichung, Taiwan; 5 Department of Psychiatry, Tsaotun Psychiatric Center, Ministry of Health and Welfare, Nantou, Taiwan; 6 Graduate Institute of Food Safety, College of Agriculture and Natural Resources, National Chung Hsing University, Taichung, Taiwan; 7 School of Gerontology Health Management & Master Program in Long-Term Care, College of Nursing, Taipei Medical University, Taipei, Taiwan; 8 Department of Physical Medicine and Rehabilitation, Taipei Medical University Hospital, Taipei, Taiwan; 9 Graduate Institute of Clinical Medicine, College of Medicine, Taipei Medical University, Taipei, Taiwan; 10 Center of Evidence-Based Medicine, Department of Education, Taipei Medical University Hospital, Taipei, Taiwan

**Keywords:** dementia, type 2 diabetes, trajectory, severe hypoglycemia, cohort studies

## Abstract

**Background:**

We aimed to investigate associations between exposure to various trajectories of severe hypoglycemic events and risk of dementia in patients with type 2 diabetes.

**Methods:**

In 2002–2003, 677,618 patients in Taiwan were newly diagnosed as having type 2 diabetes. Among them, 35,720 (5.3%) experienced severe hypoglycemic events during the 3-year baseline period following diagnosis. All patients were followed from the first day after baseline period to the date of dementia diagnosis, death, or the end of 2011. A group-based trajectory model was used to classify individuals with severe hypoglycemic events during the baseline period. Cox proportional hazard models with the competing risk method were used to relate dementia risk to various severe hypoglycemia trajectories.

**Results:**

After a median follow-up 6.70 and 6.10 years for patients with and without severe hypoglycemia at baseline, respectively, 1,952 (5.5%) individuals with severe hypoglycemia and 23,492 (3.7%) without developed dementia during follow-up, for incidence rates of 109.80 and 61.88 per 10,000 person-years, respectively. Four groups of severe hypoglycemia trajectory were identified with a proportion of 18.06%, 33.19%, 43.25%, and 5.50%, respectively, for Groups 1 to 4. Groups 3 (early manifestation but with later decrease) and 4 (early and sustained manifestation) were associated with a significantly increased risk of dementia diagnosis, with a covariate-adjusted subdistribution hazard ratio of 1.22 (95% confidence interval, 1.14–1.31) and 1.25 (95% confidence interval, 1.02–1.54), respectively.

**Conclusion:**

Our analysis highlighted that early manifestation of severe hypoglycemic events may contribute more than does late manifestation to the risk of dementia among individuals newly diagnosed as having type 2 diabetes.

## INTRODUCTION

According to an estimate by the International Diabetes Federation, the global population of individuals with diabetes is poised to rapidly increase from 415 million in 2015 to 642 million in 2040. Among age groups, the number of diabetics aged 65–79 years is set to increase more appreciably than others, by more than twofold.^1^ Because both type 2 diabetes and older age are associated with increased risk of dementia, a substantial increase in people with dementia can be expected in the future, especially in aging societies.

Recent meta-analyses of observational studies have reported that type 2 diabetes was associated with a 1.6–1.8-fold increased risk of any type of dementia, a 2.0–2.5-fold increased risk of vascular dementia, and a 1.5–2.0-fold increased risk of nonvascular dementia (mainly Alzheimer disease [AD]).^2^^,^^3^ Neuroglycopenia resulting from a hypoglycemic episode can lead to degeneration of brain cells^4^ and decline in cognitive function.^5^ Several studies have observed that experiences of hypoglycemia were associated with elevated risk of subsequent dementia in elderly people; a dose-gradient effect of recurrent hypoglycemic episodes on risk of dementia was also observed.^6^^–^^10^ Compared with people not having had a hypoglycemic episode, those with one episode had a 1.26–2.05-fold increased risk of dementia, and those with two or more episodes had a 1.50–4.07-fold increased risk.^6^^,^^7^^,^^9^^,^^10^

However, most studies have determined frequency of hypoglycemia by counting the number of hypoglycemic episodes over a period of time and associated the total number of episodes with incidence of dementia.^7^^,^^9^^,^^10^ This design presumes a constant hazard of hypoglycemic episodes over the progression of diabetes; however, this may not be accurate. One finding noted that the chance of severe hypoglycemia occurrence increased with the duration of diabetes and of insulin therapy.^11^ Moreover, it was reported that repeated episodes of severe hypoglycemia positively correlated with the presence of certain comorbidities and that a long duration of diabetes prior to insulin treatment or antihypertensive medications may reduce the risk of hypoglycemia.^12^ These findings suggest that the chance of developing hypoglycemia over the course of diabetes may be affected by both disease progression and medication.

Current evidence indicates that hypoglycemia is prevalent among people with type 2 diabetes; particularly those on insulin, but also fairly common with other treatment regimens.^13^ Because severe hypoglycemic episodes can repeatedly occur during the course of type 2 diabetes, the exposure parameters regarding severe hypoglycemia that are truly associated with elevated risk of dementia must be explored. Therefore, to better illustrate the role of severe hypoglycemia on the relationship between type 2 diabetes and dementia, we conducted a population-based study to depict the trajectory of severe hypoglycemic episodes and to explore the risk of dementia associated with various trajectories of severe hypoglycemic episodes.

## METHODS

### Data source

Data were retrieved from Taiwan’s National Health Insurance (NHI) Research Database (NHIRD), a medical claims database that contains medical records of nearly all (>97%) Taiwanese citizens (prisoner and military personnel claims were exempted during the study period).^14^ The National Health Insurance Administration conducts quarterly expert reviews on a random sample of medical claims to ensure their accuracy.^15^ Information on median family income for 368 cities and townships in 2002–2003 was retrieved from Government Open Data, which is supervised by the Taiwan National Development Council.^16^ The study was approved by the Institutional Review Board of National Cheng Kung University Hospital (A-EX-106-001).

### Research design and study cohorts

This was a claim data-based cohort study. Type 2 diabetes was identified if during 2002–2003 a patient had ≥1 admission or ≥2 ambulatory care visits for type 2 diabetes (International Classification of Diseases, Ninth Revision, Clinical Modification [ICD-9-CM]: 250.x0 or 250.x2) within a 365-day period and was not diagnosed as having type 1 diabetes (ICD-9-CM: 250.x1 or 250.x3) (*n* = 896,155). One validation study by Sung et al^17^ reported a sensitivity, specificity, positive predictive value, and negative predictive value of 90.9%, 94.9%, 92.0, and 94.2%, respectively, for the diagnosis of diabetes in Taiwan’s NHI claims if certain algorithm of case-identification can be applied. For example, both diagnosis codes and prescription medications recorded in the index hospitalization, or diagnosis codes appear in at least one inpatient, or in at least two outpatient claims in a year. The date of the first type 2 diabetes diagnosis in 2002–2003 was considered the enrollment date. Individuals with type 2 diabetes with diagnoses of severe hypoglycemic episodes between January 1, 1997, and the enrollment date were excluded (*n* = 66,507).

In determining trajectories of severe hypoglycemic episodes during the first 3 years after the enrollment date (ie, baseline period), those who died or withdrew from the NHI program during the baseline period were also excluded (*n* = 89,839). We further excluded people having been diagnosed as having dementia prior to the end of the baseline period (*n* = 62,191), leaving a total of 677,618 people with a complete 3-year baseline period in the study population. Among these, 35,720 (5.3%) experienced severe hypoglycemia during the baseline period.

### Trajectory of severe hypoglycemic episodes

Hypoglycemic events recorded in medical claims from emergency or inpatient departments were considered severe hypoglycemia. By modifying the coding algorithm proposed by Ginde et al,^18^ we included the following ICD-9-CM codes as severe hypoglycemia: 250.8, 251.0, 251.1, 251.2, 270.3, and 962.3. Among the patients with type 2 diabetes who experienced one or more severe hypoglycemic event during the baseline period, we examined, by 6-month intervals, whether an individual experienced a severe hypoglycemia episode over the 3-year baseline period. The six repeated measurements constituted the trajectory of severe hypoglycemic episodes.

We employed the group-based trajectory model (GBTM)^19^ to describe and distinguish trajectory types of occurrence of severe hypoglycemia. The GBTM method assumes several latent groups of distinct polynomial functions of repeated measurements, namely trajectories. The likelihood of observing values at each time point for each individual, given they belong to one of the groups, was estimated. By maximizing the complete likelihood for all individuals (ie, the product of these individual likelihood values), polynomial growth curves were constructed to describe the shape for each trajectory group under a given number of groupings; the membership of each individual assigned to one of the groups was also be determined.^20^^,^^21^

**Figure 1.**
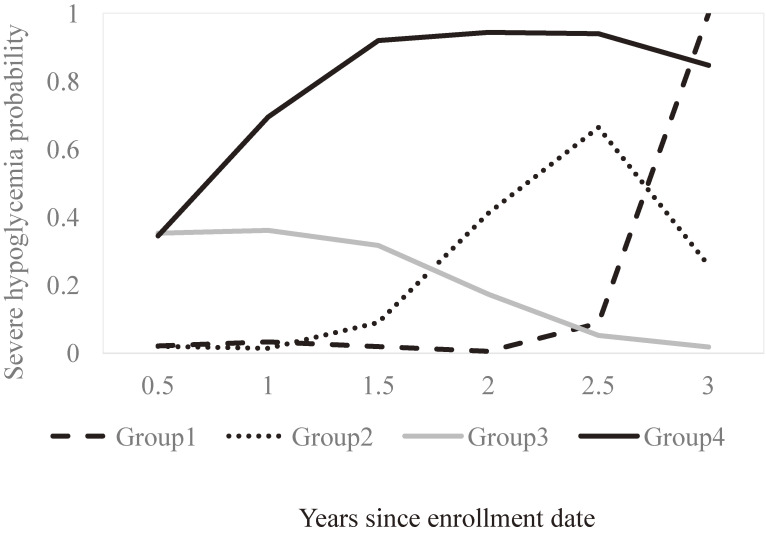
Trajectory of severe hypoglycemic episodes occurring within 3 years after enrollment. Group 1: very late manifestation (18.06% probability); Group 2: late manifestation (33.19% probability); Group 3: early manifestation with later decrease (43.25% probability); Group 4: early and sustained manifestation (5.50% probability).

In this study, we chose polynomial logistic functions to construct trajectories for occurrences of severe hypoglycemia and fitted data by linear, quadratic, and cubic functions for each trajectory. The optimal number of trajectory groups and the appropriate order of trajectories were determined by considering clinical plausibility and three statistical criteria: (1) the Bayes information criterion (BIC) of the model was minimal, (2) Bayes factors (2 log_e_ BIC_10_) between the more complex model and the simpler model exceeded 10, which is the criterion for very strong evidence against the simpler model, and (3) the group with the smallest sample size contained at least 5% of the study population.^20^ When the number and shapes of trajectory groups were determined, each individual was assigned to the group with the maximal posterior probability. By means of this group-based trajectory analysis, we classified all 35,720 individuals with type 2 diabetes into four groups with distinct trajectories of severe hypoglycemic episodes (Figure 1). According to severity, we named the groups as Group 1, very late manifestation (18.06% probability); Group 2, late manifestation (33.19% probability); Group 3, early manifestation but with later decrease (43.25% probability); and Group 4, early and sustained manifestation (5.50% probability). The mean of severe hypoglycemia episodes for Groups 1 to 4 were 1.86 (standard deviation [SD], 2.16), 3.15 (SD, 4.49), 2.38 (SD, 3.67), and 20.37 (SD, 10.94), respectively.

### Follow-up and end point

All individuals were followed from the day following the end of the baseline period (ie, index date, which was enrollment date plus 3 years) to the date of a new diagnosis of dementia, withdrawal from the NHI program, or the end of 2011. It is compulsory by law for all Taiwanese citizens and residents to register with the NHI program, and only those who leave the country permanently (eg, emigration to other countries) or die can withdraw from their NHI policies. Patients with ≥1 admission or ≥3 ambulatory care visits with a diagnosis of dementia (ICD-9-CM: 331.0 or 290.xx) during follow-up were considered to be incidences of dementia.^22^ Under Taiwan’s NHI program, neurologists or psychiatrists usually perform serum evaluation (including a complete blood count and biochemistries, iron, thyroid hormone, vitamin B_12_, folate, and syphilis), psychological examinations, and brain imaging (computed tomography or magnetic resonance imaging) to confirm the diagnosis of dementia.^23^ Taiwanese studies based on the NHI claims further used multiple diagnoses of dementia in a year to reduce the false positive rate in determining “dementia” diagnosis.^23^^,^^24^ eFigure 1 illustrates the time frame of ascertainment of newly diagnosed type 2 diabetes, the occurrence of severe hypoglycemia after type 2 diabetes, and dementia.

### Covariates

Numerous sociodemographic characteristics and clinical features were considered as covariates. Information for gender, age at enrollment, and income-based insurance premium was retrieved from the registry. The urbanization level of the area of residence for each individual was determined using the method proposed by Liu et al,^25^ who classified each township in Taiwan into seven ordered levels of urbanization according to various indicators, including population density, proportion of residents with college or higher education, percentage of elderly (>65 years) people, proportion of the workforce practicing agriculture, and number of physicians per 10^5^ people. The median annual family income of each township was also employed to represent individuals’ community socioeconomic status.

Various comorbidities that predispose a person to dementia were accounted for, including cerebrovascular disease, cardiovascular disease, diabetic microvascular disease (ie, diabetic retinal disease or end-stage renal disease), peripheral neuropathy, depression, head trauma, hypertension, and hyperlipidemia. Information regarding these comorbidities was obtained from inpatient and outpatient medical claims reimbursed between January 1, 1997, and the index date. The number of ambulatory visits during the 1-year period before enrollment was counted to indicate the frequency of clinical visits, and the frequency of clinical visits was controlled to minimize the potential of disease surveillance bias.

### Statistical methods

Count and percentage as well as mean and SD were used to describe sociodemographic characteristics and clinical features of individuals with or without severe hypoglycemia and the trajectory groups. Overall and sex- and age-stratified incidence rates for severe hypoglycemia were expressed by persons per 10,000 person-years (PYs) observed and calculated under the assumption of a Poisson distribution. We used the Cox proportional hazard regression model to estimate overall and sex- and age-specific hazard ratios (HRs) and their 95% confidence intervals (CIs) for dementia in relation to various trajectories of severe hypoglycemia occurrence during the baseline period. By taking death as a competing risk event and dementia as the risk of interest, subdistribution HRs (sHRs) were estimated based on the Fine and Gray model.^26^

Data analyses were performed using SAS (version 9.4; SAS Institute, Cary, NC, USA). A *P*-value of 0.05 or less was considered statistically significant.

## RESULTS

Compared with those without severe hypoglycemia, patients with severe hypoglycemia were older and tended to be men. They were also more likely to live in a rural area, have a lower income, make fewer clinical visits, live in a poorer community, and have comorbidities listed in Table 1. Patients in Groups 1, 2, and 3 were essentially similar with respect to the selected characteristics. However, patients in Group 4 (early and sustained manifestation) tended to be men, younger, have higher income, and were less likely to live in a rural area or have a comorbidity (Table 1).

**Table 1.** Characteristics of study diabetes population according to severe hypoglycemia status

Characteristics	Without severe hypoglycemia *n* = 641,898 *n* (%)	With severe hypoglycemia^a^				

		Overall *n* = 35,720 *n* (%)	Group 1 *n* = 6,451 *n* (%)	Group 2 *n* = 11,855 *n* (%)	Group 3 *n* = 15,449 *n* (%)	Group 4 *n* = 1,965 *n* (%)
Median follow-up time, years	6.70	6.10	5.80	6.00	6.10	6.50
Frequency of hypoglycemia						
Mean (SD)	N.A.	3.53 (6.05)	1.86 (2.16)	3.15 (4.49)	2.38 (3.67)	20.37 (10.94)
Gender						
Female	325,607 (50.73)	19,425 (54.38)	3,477 (53.9)	6,517 (54.97)	8,462 (54.77)	969 (49.31)
Male	316,291 (49.27)	16,295 (45.62)	2,974 (46.1)	5,338 (45.03)	6,987 (45.23)	996 (50.69)
Age, years						
<50	162,097 (25.25)	6,257 (17.52)	1,007 (15.61)	2,019 (17.03)	2,784 (18.02)	447 (22.75)
50–59	186,937 (29.12)	8,385 (23.47)	1,395 (21.62)	2,768 (23.35)	3,676 (23.79)	546 (27.79)
60–69	180,528 (28.12)	11,069 (30.99)	2,119 (32.85)	3,710 (31.29)	4,652 (30.11)	588 (29.92)
≥70	112,336 (17.5)	10,009 (28.02)	1,930 (29.92)	3,358 (28.33)	4,337 (28.07)	384 (19.54)
Mean (SD)	58.05 (11.98)	61.76 (11.93)	62.67 (11.70)	61.93 (11.81)	61.60 (12.08)	59.07 (11.75)
Urbanization level						
Urban area	277,431 (43.22)	12,846 (35.96)	2,394 (37.11)	4,300 (36.27)	5,398 (34.94)	754 (38.37)
Satellite area	169,739 (26.44)	9,704 (27.17)	1,725 (26.74)	3,213 (27.1)	4,223 (27.34)	543 (27.63)
Rural area	189,264 (29.49)	12,815 (35.88)	2,274 (35.25)	4,242 (35.78)	5,658 (36.62)	641 (32.62)
Income-based insurance premium (NTD)						
Dependent	121,979 (19)	7,616 (21.32)	1,360 (21.08)	2,563 (21.62)	3,321 (21.5)	372 (18.93)
0–15,000	80,632 (12.56)	5,181 (14.5)	986 (15.28)	1,729 (14.58)	2,195 (14.21)	271 (13.79)
15,000–25,000	307,341 (47.88)	18,287 (51.2)	3,312 (51.34)	6,063 (51.14)	7,946 (51.43)	966 (49.16)
>25,000	130,774 (20.37)	4,604 (12.89)	790 (12.25)	1,492 (12.59)	1,968 (12.74)	354 (18.02)
City/township family-income tertiles						
Min–Q1	154,260 (24.03)	10,387 (29.08)	1,855 (28.76)	3,493 (29.46)	4,500 (29.13)	539 (27.43)
Q1–Q3	315,283 (49.12)	17,737 (49.66)	3,183 (49.34)	5,803 (48.95)	7,788 (50.41)	963 (49.01)
Q3–Max	159,363 (24.83)	6,844 (19.16)	1,289 (19.98)	2,339 (19.73)	2,806 (18.16)	410 (20.87)
Comorbidity						
Cerebrovascular disease	99,921 (15.57)	8,500 (23.8)	1,519 (23.55)	2,850 (24.04)	3,800 (24.6)	331 (16.84)
Cardiovascular disease	286,497 (44.63)	18,887 (52.88)	3,451 (53.5)	6,294 (53.09)	8,271 (53.54)	871 (44.33)
Hypertension	384,470 (59.9)	24,409 (68.33)	4,462 (69.17)	8,196 (69.14)	10,555 (68.32)	1,196 (60.87)
Hyperlipidemia	284,276 (44.29)	15,166 (42.46)	2,719 (42.15)	5,114 (43.14)	6,521 (42.21)	812 (41.32)
Microvascular disease	37,852 (5.9)	4,768 (13.35)	897 (13.9)	1,657 (13.98)	2,041 (13.21)	173 (8.8)
Depression	39,771 (6.2)	2,589 (7.25)	449 (6.96)	837 (7.06)	1,185 (7.67)	118 (6.01)
Head trauma	35,058 (5.46)	2,762 (7.73)	492 (7.63)	881 (7.43)	1,262 (8.17)	127 (6.46)
ESRD	2,443 (0.38)	461 (1.29)	84 (1.3)	159 (1.34)	207 (1.34)	11 (0.56)

ESRD, end-stage renal disease; N.A., not applicable; NTD, New Taiwan Dollar; SD, standard deviation.

^a^Group 1: very late manifestation; Group 2: late manifestation; Group 3: early manifestation with later decrease; Group 4: early and sustained manifestation.

Over a maximum of 7 years (mean, 5.87 years) of follow-up, 1,952 (5.5%) individuals with severe hypoglycemia and 23,492 (3.7%) without were diagnosed as having dementia, corresponding to incidence rates of 109.80 and 61.88 per 10,000 PYs, respectively. The median follow-up time for patients with and without severe hypoglycemia was 6.70 and 6.10 years, respectively. Among all incident cases of dementia, 25.37% were AD (ICD-9-CM: 331.0), 11.78% were vascular dementia (VD) (ICD-9-CM: 290.4x), and the others were non-AD and non-VD. Higher incidence rates of dementia for individuals with severe hypoglycemia were observed in both men (89.01 vs 51.85 per 10,000 PYs) and women (126.85 vs 71.39 per 10,000 PYs). Among people with severe hypoglycemia in the baseline period, Group 3 (early manifestation but with later decrease) had the highest incidence rate of dementia (120.12 per 10,000 PYs), and Group 4 had the lowest incidence rate (85.94 per 10,000 PYs). Similar group discrepancy was noted for both men and women (Table 2).

**Table 2.** Incidence rates and crude subdistribution hazard ratios of dementia diagnosis in relation to groups by severity of severe hypoglycemia trajectory for patients with type 2 diabetes

	Number of people	Number of events	Person-years observed	Incidence rate (per 10,000)	Crude sHR	95% CI
Males						
Without severe hypoglycemia	316,291	9,585	1,848,634.5	51.85 (50.81–52.90)	1.00	
With severe hypoglycemia	16,295	713	80,104.9	89.01 (82.56–95.76)	1.71	(1.58–1.84)
Group 1^a^	2,974	114	13,813.3	82.53 (68.08–99.14)	1.58	(1.32–1.91)
Group 2^a^	5,338	218	25,835.4	84.38 (73.55–96.36)	1.62	(1.42–1.85)
Group 3^a^	6,987	343	34,960.9	98.11 (87.93–109.00)	1.88	(1.69–2.09)
Group 4^a^	996	38	5,495.3	69.15 (48.93–94.91)	1.33	(0.97–1.83)

Females						
Without severe hypoglycemia	325,607	13,907	1,947,998.6	71.39 (70.21–72.59)	1.00	
With severe hypoglycemia	19,425	1,239	97,673.8	126.85 (119.90–134.10)	1.77	(1.67–1.88)
Group 1	3,477	202	16,647.6	121.34 (105.20–139.30)	1.70	(1.48–1.95)
Group 2	6,517	385	32,374.9	118.92 (107.30–131.30)	1.67	(1.51–1.84)
Group 3	8,462	596	43,208.6	137.94 (127.00–149.40)	1.93	(1.77–2.09)
Group 4	969	56	5,442.7	102.89 (77.72–133.60)	1.44	(1.10–1.87)

Overall						
Without severe hypoglycemia	641,898	23,492	3,796,633.1	61.88 (61.09–62.67)	1.00	
With severe hypoglycemia	35,720	1,952	177,778.7	109.80 (105.00–114.80)	1.77	(1.69–1.85)
Group 1	6,451	316	30,460.9	103.74 (92.54–115.80)	1.67	(1.50–1.87)
Group 2	11,855	603	58,210.3	103.59 (95.45–112.20)	1.67	(1.54–1.81)
Group 3	15,449	939	78,169.5	120.12 (112.50–128.00)	1.93	(1.81–2.06)
Group 4	1,965	94	10,938.0	85.94 (69.45–105.20)	1.39	(1.13–1.70)

CI, confidence interval; sHR, subdistribution hazard ratio.

^a^Group 1: very late manifestation; Group 2: late manifestation; Group 3: early manifestation with later decrease; Group 4: early and sustained manifestation.

Table 3 presents covariate-adjusted sHRs (AsHRs) of dementia diagnosis for various groups of severe hypoglycemia trajectories. Compared with controls, both Groups 3 (early manifestation but with later decrease) and 4 (early and sustained manifestation) were associated with a significantly increased risk of dementia diagnosis, with AsHRs of 1.22 (95% CI, 1.14–1.31) and 1.25 (95% CI, 1.02–1.54), respectively. Further gender-specific analyses suggested that only Group 3 not Group 4 was significantly associated with increased risk of dementia, with AsHRs of 1.22 (95% CI, 1.09–1.36) in men and 1.23 (95% CI, 1.13–1.34) in women. Additionally, age-specific analyses suggested that Groups 1 (AsHR 1.41), 2 (AsHR 1.31), and 3 (AsHR 1.21) were all significantly associated with increased AsHR of dementia in younger (<65 years of age) patients. However, only Group 3 was significantly associated with increased AsHR of dementia for older (≥65 years) patients (AsHR 1.23).

**Table 3.** Covariate-adjusted subdistribution hazard ratios of dementia diagnosis in relation to groups of severe hypoglycemia incidence trajectory for patients with type 2 diabetes

Gender/Age, years	Without severe hypoglycemia *n* = 641,898	Groups of severe hypoglycemia trajectory^a^			

		*n* = 35,720			
		Group 1 (*n* = 6,451, 18.06%) AsHR^b,c^ (95% CI)	Group 2 (*n* = 11,855, 33.19%) AsHR^b,c^ (95% CI)	Group 3 (*n* = 15,449, 43.25%) AsHR^b,c^ (95% CI)	Group 4 (*n* = 1,965, 5.50%) AsHR^b,c^ (95% CI)

Male (*n* = 332,586)	1.00	0.90 (0.75–1.09)	1.00 (0.87–1.14)	1.22 (1.09–1.36)	1.21 (0.88–1.67)
<65 years	1.00	1.25 (0.87–1.81)	1.08 (0.81–1.45)	1.12 (0.87–1.43)	0.74 (0.33–1.66)
≥65 years	1.00	0.82 (0.66–1.01)	0.98 (0.84–1.14)	1.25 (1.11–1.42)	1.37 (0.96–1.94)

Female (*n* = 345,032)	1.00	0.98 (0.85–1.12)	1.02 (0.92–1.13)	1.23 (1.13–1.34)	1.29 (0.99–1.68)
<65 years	1.00	1.54 (1.16–2.04)	1.47 (1.20–1.82)	1.27 (1.04–1.55)	1.79 (1.11–2.88)
≥65 years	1.00	0.88 (0.75–1.03)	0.93 (0.83–1.04)	1.22 (1.11–1.34)	1.15 (0.84–1.58)

Overall (*N* = 677,618)	1.00	0.94 (0.84–1.06)	1.01 (0.93–1.10)	1.22 (1.14–1.31)	1.25 (1.02–1.54)
<65 years	1.00	1.41 (1.13–1.77)	1.31 (1.11–1.56)	1.21 (1.03–1.41)	1.31 (0.87–1.97)
≥65 years	1.00	0.85 (0.75–0.97)	0.95 (0.86–1.04)	1.23 (1.14–1.33)	1.24 (0.98–1.56)

AsHR, adjusted subdistribution hazard ratio; CI, confidence interval.

^a^Group 1: very late manifestation; Group 2: late manifestation; Group 3: early manifestation with later decrease; Group 4: early and sustained manifestation.

^b^Covariates adjusted included gender, age, urbanization level, income-based insurance premium, annual ambulatory visits, township family-income tertiles, and comorbidities (cerebrovascular disease, cardiovascular disease, hypertension, hyperlipidemia, microvascular disease, peripheral neuropathy, depression, head trauma, and end-stage renal disease).

^c^Competing risk mortality was considered by Fine and Gray’s model.

Compared to those who did not suffer from severe hypoglycemia, patients with severe hypoglycemia at baseline had higher prescriptions of various anti-glucose drugs, including sulfonylurea, meglitinide, thiazolidinedione, alpha-glucosidase inhibitor, and insulin. They also had significantly higher prevalence of beta-blocker use. We also noted differences in anti-glucose use among different groups of patients with severe hypoglycemia. Both sulfonylurea and alpha-glucosidase inhibitor were prevalent in patients of Group 4 who had a higher frequency of severe hypoglycemia. (eTable 1).

Our study used severe hypoglycemia events in the first 3 years after type 2 diabetes diagnosis to determine severe hypoglycemia trajectories and ignored any severe hypoglycemia event occurring during follow-up, which might be subject to biased estimation of dementia risk associated with severe hypoglycemia. We performed a sensitivity analysis by including severe hypoglycemia events occurring during the followed period to assess their impact on incident dementia. The results are shown in eTable 2. Similar to the original analysis, both Groups 3 and 4 (ie, patients with early manifestation of severe hypoglycemia) were still at significantly elevated risk of dementia, with an HR of 1.25 (95% CI, 1.17–1.33) and 1.36 (95% CI, 1.11–1.67), respectively. Patients in Groups 1 and 2, on the other hand, were not significantly associated with dementia. Furthermore, the severe hypoglycemia events occurring during follow-up period also posed a moderate but significant impact on incident dementia, with an HR of 1.14 (95% CI, 1.10–1.18).

## DISCUSSION

The associations between and mechanisms of hypoglycemia and dementia have been documented.^27^^–^^30^ The dose-gradient effect of hypoglycemic episodes on dementia has also been reported.^6^^,^^7^ However, to the best of our knowledge, this is the first study to investigate long-term dynamic trajectories of severe hypoglycemic episodes using the national medical claims data of the entire population of a country, the number of which may be sufficient for reliable trajectory grouping and comparison. Our findings not only provide further support for the notion that severe hypoglycemia may modestly increase the risk of dementia in people with diabetes but also highlight the potential adverse effect of early manifestation of severe hypoglycemia on subsequent risk of dementia.

Few (5.3%) patients with type 2 diabetes experienced severe hypoglycemia within 3 years after type 2 diabetes was newly diagnosed. Among them, individuals in Groups 3 and 4 had severe hypoglycemia that manifested at an earlier time during the 3-year baseline period, representing 43.25% and 5.50%, respectively, of the study cohort. Severe hypoglycemic events were sustained for the entire 3-year baseline period for Group 4 patients, who were identified as more likely to be men, younger, and living in more affluent areas. This group also had fewer comorbidities. This finding is consistent with results reported by Yen et al,^31^ who examined factors that influenced continuing care participation of patients with type 2 diabetes and factors contributing to interrupted participation of patients with diabetes enrolled in a diabetes pay-for-performance (P4P) program in Taiwan. They determined that interruption of P4P program participation occurred in 78,759 (44.33%) of the enrolled patients with diabetes and was correlated with male sex, younger age (<35 years), residence in areas with the highest urbanization levels, and greater severity of diabetes complications.

Even after adjustment for various sociodemographic characteristics and selected comorbidities, patients with early manifestation of severe hypoglycemia (ie, Groups 3 and 4) were associated with significantly increased risk of dementia. Three possible explanations may account for this. First, patients with early occurrence of severe hypoglycemic events are prone to dysglycemia (ie, fluctuation of blood sugar levels) and have a greater chance of resultant lower white and gray matter volume, as well as reduced visuospatial function and cognitive speed.^32^ Moreover, previous studies have proposed that poor glycemic controls, such as impaired HbA1C levels and fluctuation of glucose values, are associated with higher risk of hypoglycemia-related cognitive impairment among patients with type 2 diabetes.^33^^–^^35^ Second, patients newly diagnosed as diabetic with early manifestation of hypoglycemia abstracted from the medical claims may have actually had diabetes for a longer period because type 2 diabetes has a high likelihood of delayed and under-diagnosis.^36^ The International Diabetes Federation has reported that undiagnosed type 2 diabetes ranges from 24.1% to 75.1% among adult diabetes patients.^37^ Third, the baseline period was 3 years in our study design. Because Group 3 and 4 individuals developed severe hypoglycemia at an earlier time than those in Groups 1 and 2, they may be expected to have a longer duration of exposure to severe hypoglycemia, which increases the risk of dementia.^38^

We further compared Group 3 and Group 4 trajectories and noted that Group 4 had more sustained severe hypoglycemic events and represented slightly higher overall and age-specific AsHRs, although the age-specific AsHRs were revealed to be nonsignificant. Theoretically, early and persistent manifestation of hypoglycemia (eg, Group 4 patients) would demonstrate the highest risk of dementia. Animal models have demonstrated that severe hypoglycemia may induce hippocampal neuronal damage, directly resulting in learning and memory deficits.^39^^,^^40^ Other clinical studies of patients with diabetes have noted that severe hypoglycemia alters brain structure^41^ and causes significant cognitive damage.^42^^,^^43^ Hence, sustained hypoglycemic episodes are likely interrelated with dementia through the extent of neuronal damage following various forms of central nervous system insults (eg, stroke) in both preclinical^44^ and clinical^45^ stages, which may account for the significantly increased risk of dementia in Group 4 patients. The elevated but nonsignificant risk of dementia in the sex- and age-specific analyses of Group 4 patients could be due to inadequate sample size. The possible pathophysiological mechanism for the relationship between sustained severe hypoglycemic events and dementia might be that severe hypoglycemia is likely to interrelate with dementia through the counterbalance of glucose-sensing neurons between glucose-excited or glucose-inhibited neuronal activities,^46^ resulting in neuronal cell death.^38^ Therefore, early severe hypoglycemic episodes, especially with a persistent pattern, may be a novel risk factor, in addition to conventional factors, for subsequent dementia among patients with type 2 diabetes.

A noteworthy strength of this study was the novel use of severe hypoglycemia trajectories in association with the risk of dementia. Our results clearly demonstrated that not only the presence of severe hypoglycemia but also its trajectories were significantly associated with the risk of dementia, which has imminent clinical implications. Another methodological strength is that we considered death a competing risk event in our analysis, avoiding overestimation of the risk of dementia in relation to severe hypoglycemia.^47^

Despite these strengths, the algorithm used to ascertain disease might be subject to inadequate sensitivity, specificity, positive predictive value, and negative predictive value because the algorithm was solely dependent upon electronic medical records (EMR). We preferably need a high positive predictive value in identifying our cohort patients with severe hypoglycemia. In addition, a high specificity is essential for our claim data to classify dementia status.^48^ It is unfortunate that the complete information concerning the above validity-related criteria is still lacking for Taiwan’s NHI datasets. Apart from the potential problems with the use of EMR, there are still some other weaknesses that need to be discussed. First, information for blood glucose level and cognitive function was not available in the NHIRD because such information is not required for claims reimbursement. Individuals with mild hypoglycemia or mild cognitive impairment may not necessarily be identified in the medical claims data. Second, neither clinical parameters, including genotypes, HbA1C level, blood pressure, or body mass index, nor health-related behaviors related to blood sugar control such as hypoglycemia awareness, physical exercise, alcohol consumption, smoking, missed meal, or diet control were available in the NHIRD.^11^^,^^49^^–^^51^ In addition, indicators of socioeconomic status, such as educational attainment, have been documented to affect both hypoglycemia and dementia in patients with diabetes.^52^ Omitting these variables from the analyses may have resulted in a degree of residual confounding. Third, we included patients with type 2 diabetes who survived at least 3 years following the enrollment date (ie, the 3-year baseline period), which might have entailed a potential for selection bias if those who died earlier had specific trajectories of severe hypoglycemia. Fourth, although we managed to correctly identifying the events of type 2 diabetes, severe hypoglycemia, and dementia using certain algorithms, the potential for disease misclassification may not be entirely avoided. Misclassification of severe hypoglycemia was likely to be non-differential as the degree of misclassification is unlikely to be affected by dementia status, which occurred years later. Non-differential misclassification of severe hypoglycemia is likely to attenuate the association between severe hypoglycemia trajectory and dementia,^53^^,^^54^ which could contribute to the null association of dementia with both Groups 1 and 2. On the other hand, there might be a potential for detection bias in which diabetes patients with severe hypoglycemia might have a higher chance of being diagnosed as dementia due to their frequent clinical visits. To address this concern, we calculated the average annual number of inpatient/outpatient visits at the 3-year baseline period and then adjusted the average annual number of clinical visits in the regression model. The mean annual number of clinical visits for patients in Group 1 to Group 4 was 35.69 (SD, 23.09), 36.28 (SD, 23.58), 38.83 (SD, 24.87), and 33.99 (SD, 21.98), respectively, which were all higher than the figure for controls (29.86; SD, 19.25). Although the ORs of dementia associated with various trajectories of severe hypoglycemia slightly reduced, the results were essentially very similar, suggesting small magnitude of detection bias in our study. Because the ascertainment of disease from EMR could be involved in both non-differential and differential disease misclassification, it is very difficult to assess the overall influence of disease misclassification on our study findings.

### Conclusion

We demonstrated that early manifestation and uninterrupted clusters of severe hypoglycemia within a 3-year period after new diagnosis of type 2 diabetes were significantly associated with an increased risk of subsequent dementia. As such, early or persistently recurrent severe hypoglycemia can be used as a measure for risk stratification of dementia in patients with type 2 diabetes. We conclude that patients with type 2 diabetes experiencing early or persistent hypoglycemic episodes after a new diagnosis should be managed promptly not only for any underlying comorbidities, such as vascular diseases, but also for their glucose levels to reduce the subsequent risk of dementia.

## SUPPLEMENTARY MATERIAL

eFigure 1. Illustration of ascertainment of type 2 diabetes, severe hypoglycemic episode occurrence, and dementia. Case A: a patient newly diagnosed as having type 2 diabetes in 2002 but who experienced no severe hypoglycemic events during the first 3 years following diagnosis. Case A later developed dementia between the index date and the end of 2011. Case B: a patient newly diagnosed as having type 2 diabetes in 2003 and who experienced two episodes of severe hypoglycemia during the first 3 years following the diagnosis. Case B developed no dementia after the index date through the end of 2011.

eTable 1. Comparison of prevalence for anti-glucose drug and beta-blocker use at baseline between patients with and without severe hypoglycemia

eTable 2. Crude and covariate-adjusted subdistribution hazard ratios of dementia diagnosis in relation severe hypoglycemia assessed at baseline and during follow-up
