# Supplementary material for: Association Between Trajectory of Severe Hypoglycemia and Dementia in Patients With Type 2 Diabetes: A Population-based Study
Source: J Epidemiol. 2022 Sep 5;32(9):423–30. doi: 10.2188/jea.JE20200518 (PMC9359896; doi:10.2188/jea.JE20200518)
Supplement: Supplementary file 1 [file je-32-423-s001.pdf]

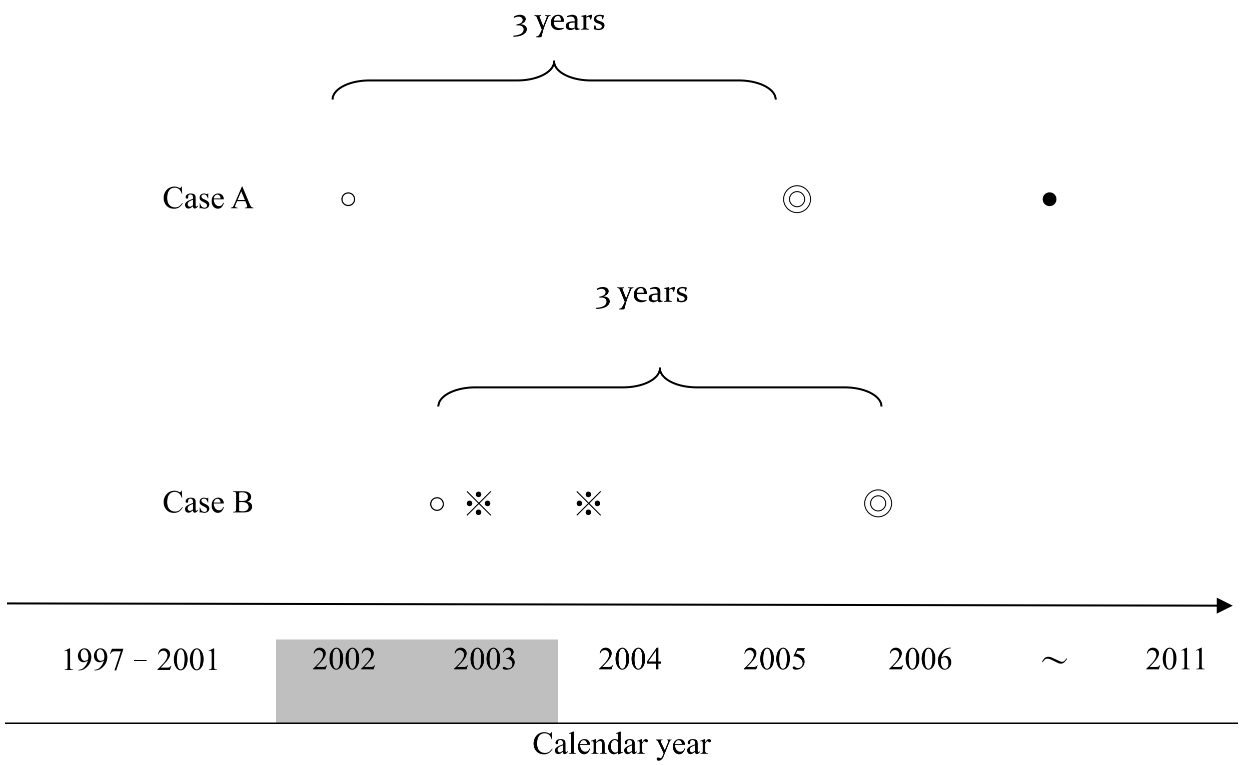

○: Enrollment date: date of newly diagnosed type 2 diabetes in 2002–2003

◎: Index date: date follow-up began (i.e., enrollment date + 3 years)

※: Severe hypoglycemic event

●: Incidence of dementia

eFigure 1. Illustration of ascertainment of type 2 diabetes, severe hypoglycemic episode occurrence, and dementia. Case A: a patient newly diagnosed as having type 2 diabetes in 2002 but who experienced no severe hypoglycemic events during the first 3 years following diagnosis. Case A later developed dementia between the index date and the end of 2011.Case B: a patient newly diagnosed as having type 2 diabetes in 2003 and who experienced two episodes of severe hypoglycemia during the first 3 years following the diagnosis. Case B developed no dementia after the index date through the end of 2011.

eTable 1. Comparison of prevalence for anti-glucose drug and beta-blocker use at baseline between patients with and without severe hypoglycemia

Drugs	Patients without severe hypoglycemia (n=641,898)	Patients with severe hypoglycemia			
		Group 1 (n=6,451)	Group 2 (n=11,855)	Group 3 (n=15,449)	Group 4 (n=1,965)
	%	%	%	%	%
Sulfonylurea					
No	26.50	16.93	14.15	23.97	6.67
Yes	73.50	80.37	85.85	76.03	93.33
Meglitinide (glinides)					
No	92.19	84.11	81.60	86.14	83.33
Yes	7.81	15.89	18.40	13.86	16.67
Thiazolidinedione					
No	85.51	81.78	76.42	83.15	76.67
Yes	14.49	18.22	23.58	16.85	23.33
Alpha-glucosidase inhibitor					
No	87.82	84.11	81.60	80.15	76.67

Yes	12.18	15.89	18.40	19.85	23.33
Insulin					
No	89.08	69.63	65.09	67.42	73.33
Yes	10.92	30.37	34.91	32.58	26.67
Metformin					
No	34.69	30.37	28.30	36.33	26.67
Yes	65.31	69.63	71.70	63.67	73.33
Beta-blocker					
No	67.65	56.54	65.09	63.30	60.00
Yes	32.35	43.46	34.91	36.70	40.00
Total	100.00	100.00	100.00	100.00	100.00

eTable 2. Crude and covariate-adjusted subdistribution hazard ratios of dementia diagnosis in relation severe hypoglycemia assessed at baseline and during follow-up

	Crude sHR[a]	95% CI	Adjusted sHR[a, b]	95% CI
Without severe hypoglycemia (n=641 898)	1.00		1.00	
With severe hypoglycemia at baseline				
Group 1 (n=6,451)	1.67	(1.50–1.87)	0.98	(0.88–1.10)
Group 2 (n=11,855)	1.67	(1.54–1.81)	1.04	(0.96–1.13)
Group 3 (n=15,449)	1.93	(1.81–2.06)	1.25	(1.17–1.33)
Group 4 (n=1,965)	1.39	(1.13–1.70)	1.36	(1.11–1.67)
With severe hypoglycemia during follow-up period	1.29	(1.25–1.33)	1.14	(1.10–1.18)

CI, confidence interval; sHR, subdistribution hazard ratio.

[a] Covariates adjusted included gender, age, urbanization level, income-based insurance premium, annual ambulatory visits, township family-income tertiles, and comorbidities (cerebrovascular disease, cardiovascular disease, hypertension, hyperlipidemia, microvascular disease, peripheral neuropathy, depression, head trauma, and end-stage renal disease)

[b] Competing risk mortality was considered by Fine and Gray's model
